# Inhibition of the Neuronal Calcium Sensor DREAM Modulates Presenilin-2 Endoproteolysis

**DOI:** 10.3389/fnmol.2018.00449

**Published:** 2018-12-03

**Authors:** Rocío Naranjo, Paz González, Alejandro Lopez-Hurtado, Xosé M. Dopazo, Britt Mellström, José R. Naranjo

**Affiliations:** ^1^Spanish Network for Biomedical Research in Neurodegenerative Diseases (CIBERNED), ISCIII, Madrid, Spain; ^2^National Biotechnology Center (CNB), CSIC, Madrid, Spain

**Keywords:** presenilins, calcium, DREAM, neuronal calcium sensors, repaglinide

## Abstract

Deregulated intracellular Ca^2+^ and protein homeostasis underlie synaptic dysfunction and are common features in neurodegenerative diseases. DREAM, also known as calsenilin or KChIP-3, is a multifunctional Ca^2+^ binding protein of the neuronal calcium sensor superfamily with specific functions through protein-DNA and protein-protein interactions. Small-molecules able to bind DREAM, like the anti-diabetic drug repaglinide, disrupt some of the interactions with other proteins and modulate DREAM activity on Kv4 channels or on the processing of activating transcription factor 6 (ATF6). Here, we show the interaction of endogenous DREAM and presenilin-2 (PS2) in mouse brain and, using DREAM deficient mice or transgenic mice overexpressing a dominant active DREAM (daDREAM) mutant in the brain, we provide genetic evidence of the role of DREAM in the endoproteolysis of endogenous PS2. We show that repaglinide disrupts the interaction between DREAM and the C-terminal PS2 fragment (Ct-PS2) by coimmunoprecipitation assays. Exposure to sub-micromolar concentrations of repaglinide reduces the levels of Ct-PS2 fragment in N2a neuroblastoma cells. These results suggest that the interaction between DREAM and PS2 may represent a new target for modulation of PS2 processing, which could have therapeutic potential in Alzheimer’s disease (AD) treatment.

## Introduction

The neuronal calcium sensor DREAM, also known as calsenilin or KChIP-3, is a multifunctional Ca^2+^ binding protein with specific roles in different subcellular compartments through protein-DNA and/or protein-protein interactions. Thus, in the nucleus DREAM binds to DRE sites in the DNA and represses transcription of target genes (Carrión et al., 1999). In addition, DREAM interacts with other nucleoproteins, like CREB, CtBP1, nuclear receptors or TTF-1 and modifies their transcriptional function (Ledo et al., 2002; Rivas et al., 2004; Scsucova et al., 2005; Zaidi et al., 2006). Outside the nucleus, the DREAM interactome comprises a heterogeneous set of proteins encompassing ion channels, membrane receptors, GRK kinases and presenilins (PSs), among others (reviewed in Rivas et al., 2011; Burgoyne and Haynes, 2012). Binding of DREAM to these proteins modulates channel gating, response to agonist stimulation (An et al., 2000; Savignac et al., 2005; Rivas et al., 2009; Wu et al., 2010) or regulates DREAM activity through post-translational modifications including phosphorylation or sumoylation (Ruiz-Gomez et al., 2007; Palczewska et al., 2011). Binding to Ca^2+^ induces conformational changes in DREAM that prevents DREAM binding to DRE sites in the DNA (Carrión et al., 1999) and distinctly modifies the interaction with other proteins (Holmqvist et al., 2001; Naranjo et al., 2016). Furthermore, binding to arachidonic acid and other small-molecules, like repaglinide or Cl-888, also induces changes in DREAM that affect its activity on Kv4 channel gating or on the processing of activating transcription factor 6 (ATF6; Holmqvist et al., 2001; Okada et al., 2003; Bowlby et al., 2005; Naranjo et al., 2016).

PSs (PS1 and PS2) are the catalytic core of the γ-secretase complex, an enzymatic activity linked to the plasma membrane that cleaves multiple intramembrane substrates including the amyloid precursor protein (APP), cell surface receptors and adhesion molecules like Notch, E-cadherin and ErbB4 (McCarthy et al., 2009; Haapasalo and Kovacs, 2011). In addition, PS proteins participate in other γ-secretase independent cellular functions including Wnt/β-catenin signaling, calcium homeostasis, apoptosis, protein trafficking, lysosomal function and autophagy (reviewed in Jurisch-Yaksi et al., 2013; Duggan and McCarthy, 2016). Importantly, mutations in the PS genes are associated with inherited familial Alzheimer’s disease (AD), and they influence both γ-secretase dependent and independent cellular functions (Sherrington et al., 1995; Zhang et al., 1998; Lee et al., 2010; Supnet and Bezprozvanny, 2011).

Posttranslational modifications of the PSs include endoproteolysis, caspase cleavage, phosphorylation and ubiquitination (Duggan and McCarthy, 2016). PS endoproteolysis occurs in the large cytosolic loop of the protein generating N- and a C-terminal fragments that remain stably associated as a heterodimer, which constitutes the active core of the γ-secretase holoenzyme (Wolfe et al., 1999; Esler et al., 2000; Li et al., 2000; Spasic and Annaert, 2008; Lu et al., 2014; Sun et al., 2015). PS proteins are highly homologous, however, fragments generated from either PS1 or PS2 do not interact with each other, suggesting that the two PSs form independent complexes (Capell et al., 1998; Saura et al., 1999). Further supporting the idea of independent functions for PS proteins, PS1 knockout (KO) mice die shortly after birth displaying aberrant defects in the central nervous system and spinal ganglia (Shen et al., 1997; Wong et al., 1997). To date, the protease activity that mediates PS proteolysis is unknown, though the acidic protease inhibitor pepstatin A is the best known PS endoproteolysis inhibitor (Campbell et al., 2002). The involvement of other protease activities in PS cleavage like the proteasome (Liu et al., 2003; Kopan and Ilagan, 2004; Massey et al., 2005) or the γ-secretase complex (Takasugi et al., 2003; Xie et al., 2005; Fukumori et al., 2010; Honarnejad et al., 2013) has been suggested. Importantly, PS endoproteolysis is developmentally regulated (Hartmann et al., 1997) and the amount of PS fragments in the cell is tightly controlled (Steiner et al., 1998; Saura et al., 1999), though the mechanisms that regulate these activities are not well understood. Other posttranslational changes are also important for PS function. Thus, PS1 phosphorylation occurs at multiple sites by a variety of kinases and modulates γ-secretase activity (Kuo et al., 2008), γ-secretase independent functions (Walter et al., 1999; Uemura et al., 2007) and PS stability (Lau et al., 2002). Moreover, caspase cleavage of the C-terminal fragment of PS2 (Ct-PS2) has been associated with the role of PS2 in apoptosis (Alves da Costa et al., 2002, 2003, 2006) and PS ubiquitination has been related to the stability of the full-length holoproteins and the degradation of the PS fragments (Massey et al., 2005).

Yeast two-hybrid assays identified the interaction between Ct-PS2 and DREAM, and coimmunoprecipitation experiments after heterologous overexpression in COS-7 cells confirmed the binding of DREAM to PS1 and PS2 C-terminal fragments (Buxbaum et al., 1998). Recently, the structural determinants of the DREAM-Ct-PS1 interaction have been elegantly analyzed (Pham and Miksovska, 2016). Upon binding, DREAM regulates PS activity at different levels. First, overexpression of DREAM in human neuroglioma H4 cells increases PS2 cleavage and accumulation of Ct-PS2 (Buxbaum et al., 1998). Since mutations in the PS genes are associated with the development of familial type of AD (FAD), this finding linked DREAM/calsenilin and neurodegeneration in AD (Buxbaum et al., 1998). This mechanism, however, was questioned in a more recent study that showed no change or a decrease in Ct-PS2 in human neuroblastoma SH-5YSY cells overexpressing DREAM or Ca^2+^-insensitive DREAM mutants, respectively (Fedrizzi et al., 2008). Second, binding of PS2 to DREAM increases γ-secretase activity and the levels of Aβ peptides, one of the end-products of APP processing. In this regard, genetic ablation of DREAM leads to reduced APP processing and lower brain levels of Aβ peptides (Lilliehook et al., 2003). Third, PSs regulate calcium homeostasis through interaction with the Sarco/ER/Ca^2+^-ATPase (SERCA) pump, InsP_3_ and ryanodine receptors and/or forming leak channels for calcium ions in the endoplasmic reticulum membrane (Tu et al., 2006; Cheung et al., 2008; Green et al., 2008; Hayrapetyan et al., 2008; Nelson et al., 2011). DREAM overexpression enhances the depletion of calcium from the ER store (Fedrizzi et al., 2008) and reduction of PS endoproteolysis reduces calcium release from this subcellular compartment (Honarnejad et al., 2013). Finally, PSs have been associated with apoptosis through γ-secretase-dependent and -independent pathways (Alves da Costa et al., 2002, 2003, 2006; Wang et al., 2005; Zeng et al., 2015) and DREAM overexpression have been associated with cell death (Jo et al., 2001). Whether DREAM-induced Ct-PS2 fragment accumulation mediates this effect is currently not established.

Here, we show that endogenous DREAM interacts with PS2 and regulates PS2 endoproteolysis in the brain. Moreover, repaglinide, a DREAM binding molecule, blocks the DREAM/PS2 interaction and reduces PS2 processing in N2a mouse neuroblastoma cells.

## Materials and Methods

### Mice

Homozygous DREAM KO mice (Cheng et al., 2002) and transgenic mice expressing a dominant active mutant DREAM insensitive to calcium (Dierssen et al., 2012; Mellström et al., 2014) were used to assess the role of DREAM in PS2 endoproteolysis in the brain.

### Cells

N2a mouse neuroblastoma and HEK293T cells were from ATCC. All cells were cultured in DMEM (with 10% FBS, penicillin/streptomycin, Glutamax; all from Invitrogen). Cell cultures were routinely checked for mycoplasma infection. To evaluate the effect of DREAM binding molecules on PS processing, neuroblastoma cells were exposed to increasing concentrations of repaglinide (30 nM to 3 μM, Sigma) and harvested 36 h later.

### Coimmunoprecipitation

Endogenous DREAM and PS2 proteins were coimmunoprecipitated from whole cell extracts (200 μg) from mouse brain or N2a cells, using 5 μg affinity-purified mouse monoclonal anti-DREAM 1B1 (Ledo et al., 2002) Tagged proteins were coimmunoprecipitated from whole cell extracts (200 μg) from HEK293T cells cotransfected with plasmids encoding Myc-DREAM_71–256_ and Flag-Ct-PS2_335–448_, using 1 μg affinity-purified rabbit anti-Myc (ab9106, Abcam) in the presence of vehicle (DMSO) or repaglinide. Immunoprecipitated PS2 was detected by western blot with anti-PS2 (Cell Signaling Technology, #2192; endogenous) or mouse anti-Flag antibody (Sigma; overexpressed).

### Western Blot

Mouse hippocampal and cortical extracts were prepared as described (López-Hurtado et al., 2018). Briefly, brain tissue was homogenized on ice in lysis buffer (RIPA, Cell Signaling Technology, #9806) supplemented with 1 mM PMSF. After 20 min incubation on ice, extracts were cleared by centrifugation (14,000× *g*, 20 min) and protein concentration assessed (Bradford, BioRad). For the analysis of the effect by DREAM binding molecules on PS2 processing, N2a cells were treated with repaglinide, or vehicle (DMSO) at the indicated concentrations for 36 h. Cells were pelleted and incubated on ice in lysis buffer for 45 min, with occasional vortexing. Samples (20 μg) were analyzed by SDS-PAGE and immunoblot. PVDF membranes were incubated overnight at 4°C with rabbit anti-PS2 (Cell Signaling Technology, #2192 or Abcam, EP1515Y, ab51249). Secondary antibody used was HRP-conjugated donkey anti-rabbit IgG (Jackson) and signals were detected with ECL Select (GE Healthcare). Control for equal loading were with total protein measured by Coomassie staining of the membrane after immunoblotting or HRP-conjugated β-actin (Sigma, A3854 clone AC-15). Lane and band intensity were quantified with ImageLab software (BioRad). We obtained similar results when we used as a primary antibody a rabbit monoclonal antibody raised against a C-terminal peptide of PS2 (Abcam Ab51249). This confirmed the specificity of the Ct-PS2 band detected by western blot.

### Real-Time Quantitative PCR

RNA was isolated from tissue samples using TRIzol, treated with DNase (Ambion) and reverse transcribed using hexamer primer and Moloney murine leukemia virus reverse transcriptase. To confirm the absence of genomic DNA, each sample was processed in parallel without reverse transcriptase. Real-time quantitative PCR (qPCR) for endogenous PS2 was performed using the assay from Applied Biosystems (Mm00448405_m1) and expression was normalized using HPRT as reference with primers 5′ TTG GAT ACA GGC CAG ACT TTG TT 3′ and 5′ CTG AAG TAC TCA TTA TAG TCA AGG GCA TA 3′ and the MGB probe 5′ TTG AAA TTC CAG ACA AGT TT 3′.

### Statistical Analysis

All data values shown are mean ± SEM. Differences were considered significant at *P* < 0.05. When possible, ordinary one-way ANOVA was used to analyze statistical differences among groups. For small or unequal sample size or non-Gaussian distribution, comparisons between groups were analyzed using *t*-test or the nonparametric ANOVA, Kruskal-Wallis test with Dunn’s multiple comparison between groups. Animal experiments were randomized. Sample size was not predetermined by statistical method. Prism 6.0 software (GraphPad) was used for statistical analysis.

### Study Approval

Experiments with mice were conducted in accordance with standard ethical guidelines (European Communities Directive 86/609 EEC; National Institutes of Health 1995). The CNB-CSIC and Madrid Community ethical committees approved the experiments with mice (PROEX 28/05).

## Results

### DREAM Regulates the Processing of Presenilin-2 in Mouse Brain

**Figure 1:**
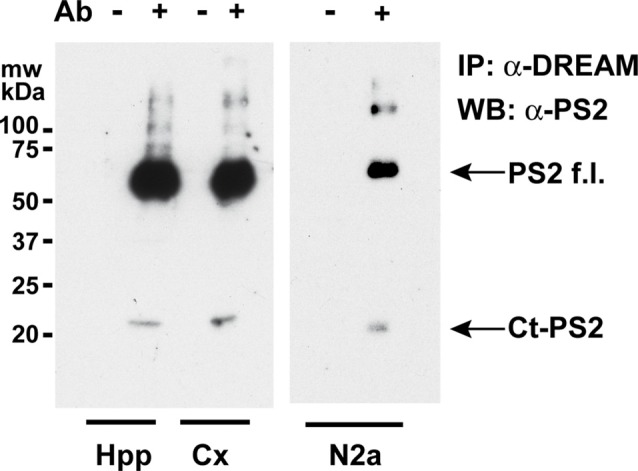
Coimmunoprecipitation of endogenous DREAM and presenilin-2 (PS2) proteins. Western blots of immunoprecipitated PS2 from mouse hippocampal (Hpp) and cortical (Cx) extracts (left) or from N2a whole cell extracts (right), with (+) or without (−) an anti-DREAM antibody (Ab), and developed with an antibody against PS2. Bands corresponding to the Ct-PS2 fragment and the full length PS2 holoprotein (PS2f.l.) are shown by arrows.

Overexpression of DREAM increased PS2 processing and the levels of the Ct-PS2 in human H4 neuroglioma but not in SH-SY5Y neuroblastoma cells (Buxbaum et al., 1998; Fedrizzi et al., 2008). Overexpression conditions could be part of the controversy between these results. Thus, we aimed to investigate whether or not endogenous DREAM regulates PS2 processing *in vivo* in basal conditions in mouse brain. Using total extracts from mouse hippocampus or cerebral cortex, coimmunoprecipitation experiments showed the interaction of endogenous DREAM and Ct-PS2 both in the hippocampus and the cerebral cortex (Figure 1). Moreover, endogenous DREAM was shown to interact not only with the Ct-PS2 processed fragment but also with the full length PS2 holoprotein (Figure 1). Similar results were observed using whole cell extracts from mouse N2a neuroblastoma cells (Figure 1).

**Figure 2:**
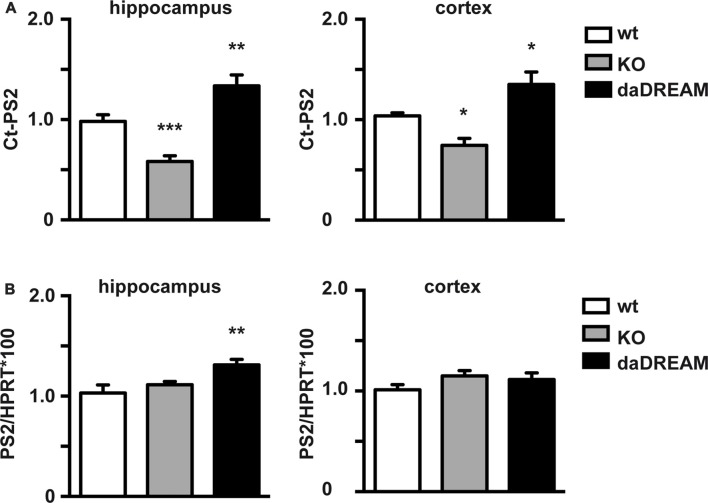
Effect of endogenous DREAM levels on PS2 endoproteolysis and PS2 gene expression. **(A)** Quantified Western blots with anti-PS2 antibody of hippocampal (Hpp) and cortical (Cx) extracts from wild-type (WT), DREAM deficient knockout (KO) and transgenic mice overexpressing dominant active DREAM (daDREAM). **(B)** Quantitative PCR (qPCR) analysis of PS2 mRNA levels in the hippocampus and the cerebral cortex from the same three genotypes as in **(A)**. Ordinary one-way ANOVA with Holm-Sidak’s multiple comparison test (*n* = 11, for each genotype) *P < 0.05, **P < 0.01, ***P < 0.001.

We then investigated whether PS2 processing is regulated by DREAM in mouse brain. For this, we compared Ct-PS2 basal brain levels in wild type (WT), DREAM deficient and transgenic mice overexpressing a dominant active DREAM (daDREAM) mutant in the brain. Absence of DREAM in DREAM deficient mice significantly reduced Ct-PS2 levels both in the cerebral cortex and the hippocampus (Figure 2A). Correspondingly, increased levels of DREAM protein in daDREAM transgenic mouse brain resulted in significant accumulation of Ct-PS2 in both brain areas (Figure 2A). Changes in Ct-PS2 levels in the cerebral cortex were not accompanied by significant changes in PS2 gene expression (Figure 2B). However, a slight but significant increase in PS2 mRNA level was observed in the hippocampus of daDREAM mice (Figure 2B). Taken together, these results indicate that endogenous DREAM interacts with endogenous PS2 and regulates PS2 endoproteolysis, most likely, without directly affecting PS2 gene expression.

### Repaglinide Blocks the DREAM-PS2 Interaction

**Figure 3:**
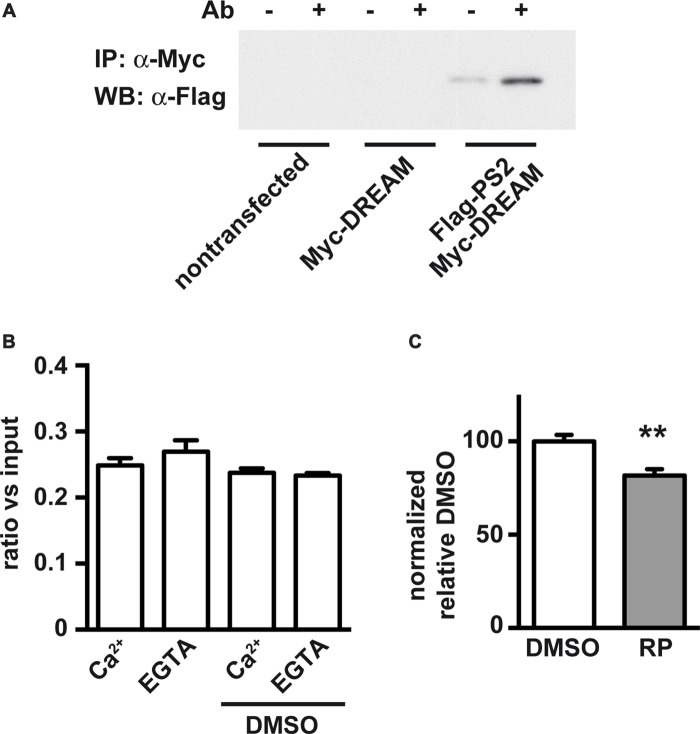
Coimmunoprecipitation of processed carboxy-terminal fragment of PS2 (Ct-PS2) and effect of calcium and the DREAM-binding molecule repaglinide. HEK293T cells were cotransfected with expression vectors for Myc-DREAM and Flag-Ct-PS2. **(A)** Immunoprecipitation with (+) or without (−) anti-Myc and immunoblot with anti-Flag antibody. **(B)** Immunoprecipitation in the presence or absence of calcium or DMSO, quantified after immunoblot with anti-Flag antibody. The experiment was repeated twice. No differences were observed among groups. **(C)** Immunoprecipitation in the presence of vehicle (DMSO) or repaglinide (RP), quantified after immunoblot with anti-Flag antibody. Unpaired two-tailed *t*-test (*n* = 10), **P < 0.01.

We next analyzed whether DREAM-binding molecules could affect the DREAM/PS2 interaction. For this, we reproduced previous coimmunoprecipitation experiments (Buxbaum et al., 1998) using HEK293T cells overexpressing tagged proteins. Combined overexpression of Myc-DREAM and Flag-Ct-PS2 resulted in coimmunoprecipitation of Flag-Ct-PS2 with an anti-Myc antibody (Figure 3A). Detection of the Flag-Ct-PS2 fragment with an anti-Flag antibody or an anti-Ct-PS2 antibody rendered identical results. As negative controls, non-transfected cells or cells transfected with only one of the expression plasmids did not show immunoprecipitation (Figure 3A). Inclusion in the immunoprecipitation reaction of 1 mM CaCl_2_ or up to 2 mM EGTA did not affect the interaction and did not modify the intensity of the immunoprecipitated band (Figure 3B). These results confirm the Ca^2+^-independent nature of this interaction, as previously described (Buxbaum et al., 1998; Fedrizzi et al., 2008). Then, we proceeded to investigate a potential blockage of the DREAM-PS2 interaction by the DREAM binding molecule repaglinide, known to affect the interaction of DREAM with other proteins including Kv4 potassium channels and ATF6 (Naranjo et al., 2016; López-Hurtado et al., 2018). *In vitro* exposure to repaglinide significantly reduced the Myc-DREAM/Flag-Ct-PS2 coimmunoprecipitation (Figure 3C). Inclusion of DMSO, the vehicle for repaglidine, as a negative control did not change the extent of the DREAM-PS2 interaction (Figure 3B).

### Repaglinide Blocks PS2 Endoproteolysis in N2a Cells

**Figure 4:**
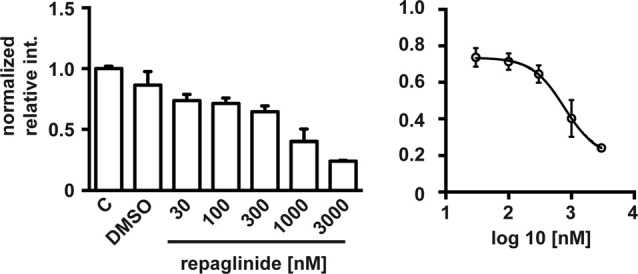
Repaglinide reduces endoproteolysis of endogenous PS2 in N2a cells. Cultures were untreated (C), treated with vehicle (DMSO) or with increasing concentrations of repaglinide. Whole cell extracts were analyzed by immunoblot with anti-PS2 antibody and band intensities quantified (left). The IC50 value (0.76 μM) was estimated after non-linear regression analysis (log(antagonist) vs. response (four parameters)) of the resulting dose-response curve (right; *n* = 2–5).

Finally, we analyzed whether partial block of the DREAM-PS2 interaction by repaglinide was translated in a noticeable effect on PS2 processing in an *in vivo* cell model. For this, we used N2a neuroblastoma cells, a cell model widely used to analyze PS2 endoproteolysis (Thinakaran et al., 1996). Exposure to repaglinide produced a concentration-dependent decrease in the levels of the Ct-PS2 fragment in N2a neuroblastoma cells with an estimated IC50 value of 0.76 μM (Figure 4).

## Discussion

In previous work, we have shown that reduction in DREAM protein levels or blockade of DREAM activity, using repaglinide, activates ATF6 processing which results in a neuroprotective effect in murine models of Huntington’s disease, delaying the onset and the progression of motor and cognitive decline in these mice (Naranjo et al., 2016; López-Hurtado et al., 2018). This finding opened a new avenue toward the search for effective HD treatments. Whether small-molecules binding to DREAM could have an effect on modifying the onset and/or the progression of other neurodegenerative pathologies is presently uncharacterized. DREAM has been associated with the progression of AD through its interaction with PSs (Buxbaum et al., 1998; Lilliehook et al., 2003). Therefore, we analyzed whether repaglinide has an effect on this interaction. Here, we show that blockade of the DREAM-PS2 interaction with repaglinide is directly translated in a decrease in PS2 endoproteolysis and in reduced levels of the Ct-PS2 fragment in N2a neuroblastoma cells. Whether this effect also occurs *in vivo* in mouse brain and the potential consequences for the onset and progression of AD remains to be investigated.

Repaglinide was developed as a potent insulinotropic agent for treatment of type-2 diabetes (Malaisse, 1995). Repaglinide binding to neuronal calcium sensors (NCS) was first reported in bovine brain and retinal extracts, which showed Ca^2+^-dependent binding respectively to neurocalcin and VILIP-1, or to recoverin (Okada et al., 2003). Repaglinide also binds to members of the DREAM/KChIP subfamily (Naranjo et al., 2016) which indicates that repaglinide binding is a characteristic of all proteins of the NCS superfamily but not to other Ca^2+^-binding proteins, including calmodulin or proteins of the S-100 superfamily (Okada et al., 2003). Nonetheless, using tagged proteins in the coimmunoprecipitation experiments here we show that repaglinide specifically blocks the DREAM-PS2 interaction. Furthermore, the interaction of PS2 with other NCS except DREAM has not been reported.

After binding, repaglinide interferes with the biological activity of the Ca^2+^ sensor, e.g., blockage of recoverin-mediated inhibition of rhodopsin kinase activity (Okada et al., 2003) DREAM-induced suppression of ATF6 processing (Naranjo et al., 2016) or, in this case, DREAM-induced activation of PS2 endoproteolysis. Work in progress, using single site mutations in DREAM aims to identify the binding pocket for repaglinide in the DREAM protein and whether these point mutations affect the interaction with PS2 and/or the induction of PS2 proteolysis.

The mechanisms that control PS endoproteolysis and finely keep a stoichiometry approximately 1:1 between N- and C-terminal fragments are poorly understood. To further complicate the picture, posttranslational mechanisms that potentially participate in these processes might be different for PS1 and PS2. Phosphorylation of PS1, but not of PS2, is known to occur at multiple sites within the cytosolic loop involving multiple kinases (Duggan and McCarthy, 2016). Early *in vitro* experiments described the interaction between DREAM and the C-terminal fragments of PS1 and PS2 as well as the increase in Ct-PS2 levels upon DREAM overexpression (Buxbaum et al., 1998). Our results confirm the DREAM-PS2 interaction *in vivo* and show the effect of DREAM inhibition on Ct-PS2 levels in N2a neuroblastoma cells. The functional consequences of the DREAM-PS1 interaction in terms of PS1 endoproteolysis and, the potential effect of DREAM interacting molecules in PS1 processing remains to be analyzed.

Overexpression of PS2 or DREAM has been associated with cell death (Janicki and Monteiro, 1997; Jo et al., 2001) and this effect is further enhanced by coexpression of DREAM and PS2 but not by a truncated form of PS2 (PS2/411stop) that lacks the C-terminal part (Jo et al., 2001). A parallel increase in Aβ_1–42_ levels after coexpression of DREAM with PS2, but not with PS2/411stop, indicated the involvement of γ-secretase activation in the cell death process, however, γ-secretase independent mechanisms can not be excluded. Interestingly, interaction between the calcium binding protein calmyrin and the cytosolic loop of PS2, and with lower affinity for the same domain in PS1, has been reported (Stabler et al., 1999). Like in the case of DREAM, coexpression of calmyrin and PS2 in HeLa cells modified the subcellular distribution of these proteins and increased cell death (Stabler et al., 1999). It was not analyzed, however, whether calmyrin modifies PS2 endoproteolysis, neither whether coexpression with PS1 also increases apoptosis (Stabler et al., 1999). The calmyrin-PS2 interaction requires and is regulated by Ca^2+^, suggesting that changes in cellular calcium homeostasis might control the functional output of this interaction (Stabler et al., 1999; Zhu et al., 2004). In contrast, the DREAM-PS2 interaction has been shown to be Ca^2+^-independent (Buxbaum et al., 1998; Fedrizzi et al., 2008). Our results support this view, however, titration curves for the binding of Apo-DREAM or Ca^2+^DREAM to peptides derived from the cytosolic loop of PS1 indicated that the interaction is stronger in the presence of Ca^2+^ (Pham and Miksovska, 2016). The different experimental conditions may account for this discrepancy.

With respect to the mechanism by which DREAM regulates PS2 endoproteolysis nothing has been reported. One possibility, among others, is that DREAM could compete with ubiquilin proteins for binding to the cytosolic loop region and/or to the C-terminal region of PS2, increasing the endoproteolysis of PS2 and/or the half-life of the Ct-PS2 fragment. It has been shown that ubiquilin-1 and -2 proteins bind to PSs and regulate PS stability by two different mechanisms (Mah et al., 2000). One, it has been shown that ubiquilin-1 and -2 regulate PS endoproteolysis and overexpression of ubiquilin proteins increases the amount of full length PS2 holoprotein and reduces the formation of Nt- and Ct-PS2 fragments (Mah et al., 2000; Massey et al., 2005). Though, a more recent study from the same group suggests that ubiquilin rather increases PSs biosynthesis (Ford and Monteiro, 2007). Two, binding of ubiquilin to the PS2 was initially associated with the ubiquitin-mediated degradation of PS2 through the proteosome pathway (Massey et al., 2004, 2005), however, binding of ubiquilin to Ct-PS2 does not require ubiquitination of critical lysine residues in this domain (Ford and Monteiro, 2007). Whether DREAM increases Ct-PS2 levels *in vivo* by competing the ubiquilin/PS2 interaction will need future experimental analysis.

Taken together, the effect of repaglinide blocking the DREAM-PS2 interaction and reducing PS2 endoproteolysis suggests that the interaction between DREAM and PS2 may represent a new target for modulation of PS2 processing, which could have therapeutic potential in AD treatment. Future studies, however, should analyze whether the reduction in PS2 endoproteolysis by DREAM binding molecules efficiently translates in a decrease in γ-secretase activity, in a reduction in β-amyloid accumulation and amyloid plaque formation and, finally, in an improvement in cognition.

## Data Availability

All datasets generated for this study are included in the manuscript.

## Author Contributions

RN, PG, AL-H and XD performed the experiments and analyzed the data. BM and JRN conceived the study, analyzed the data and wrote the article.

## Conflict of Interest Statement

The authors declare that the research was conducted in the absence of any commercial or financial relationships that could be construed as a potential conflict of interest.
